# Editorial: Clinical Psychometrics: Old Issues and New Perspectives

**DOI:** 10.3389/fpsyg.2019.00947

**Published:** 2019-05-07

**Authors:** Michela Balsamo, Marco Innamorati, Dorian A. Lamis

**Affiliations:** ^1^Department of Psychological, Health, and Territorial Sciences, “G. d'Annunzio” University of Chieti-Pescara, Chieti, Italy; ^2^Department of Human Science, Università Europea di Roma, Rome, Italy; ^3^Emory University School of Medicine, Atlanta, GA, United States

**Keywords:** psychological testing, psychometrics, clinical assessment, quantitative measurement, psychological assessment

Clinical Psychometrics is defined as a discipline that deals with the definition and measurement of clinical constructs. It focuses on the theory of measurement, the construction and validation of psychological measures, and their application in the assessment of individual differences. Therefore, Clinical Psychometrics is an applied discipline, which uses psychometric tools in order to develop evidence-based procedures aimed at understanding and improving the psychological well-being of individuals.

Clinical Psychometrics can be considered as an essential tool in many fields of research related to psychological and psychiatric interventions: for example, it is useful for diagnostic assessment (in various fields, including clinical and forensic areas), and for the design and evaluation of specific psychological and pharmacological treatments.

In the Research Topic “Clinical Psychometrics: Old Issues and New Perspectives,” we were interested in disseminating a culture of integration between the “psychometric model” and the “clinical model,” promoting a scientific debate around existing measures and methods, and proposing new methods capable of combining clinical significance with quantitative rigor (Balsamo et al., 2015a,b).

Therefore, we brought together, within this research topic, contributions from researchers investigating factor invariance of new and existing instruments for measuring clinical variables; research studies developing more refined instruments for the evaluation of clinical dimensions; as well as research studies evaluating methodological issues involved in therapeutic outcomes and processes.

## Investigating the Measurement Invariance of New and Existing Instruments for Measuring Clinical Variables

An area of interest in this Research Topic was the investigation of factor invariance of psychological tests and questionnaires (e.g., Saggino et al., 2017). In fact, psychological tests are frequently administered to different populations and ethnic groups without ever testing the assumption that scores are comparable and interpretable when tests are administered to males and females, adolescents and late adults, or different populations (Balsamo et al., 2015a, 2016, 2018). As reported in “Consequences of disregarding metric invariance on diagnosis and prognosis using psychological tests,” this assumption could have severe consequences when using psychological measures in clinical contexts. In this simulation study, the authors have shown that the lack of measurement invariance can lead in different samples to over-diagnose a measured condition or diagnose it randomly without any consideration about its real presence.

In this Research Topic, papers directly tested measurement invariance of several questionnaires. For example, two articles investigated factor invariance across sex of two different instruments assessing anxiety severity (“Testing factor structure and measurement invariance across gender with Italian Geriatric Anxiety Scale)”; (“Dimensions of anxiety, age, and gender: assessing dimensionality and measurement invariance of the State-Trait for Cognitive and Somatic Anxiety (STICSA) in an Italian sample)”. Moreover, in another paper (“Psychometric Properties and Measurement Invariance of the Brief Symptom Inventory-18 Among Chinese Insurance Employees”), the authors investigated factor invariance for the Brief Symptom Inventory-18, a common screening tool for psychological symptoms. Lastly, the paper “Is parent–child disagreement on child anxiety explained by differences in measurement properties? An examination of measurement invariance across informants and time” aimed to longitudinally investigate measurement invariance between maternal and child reports across ages in anxiety assessment. The authors moved from the evidence that agreement between parent-reports of youth and youth self-reports of anxiety problems is modest at best and demonstrated that inter-informant agreement could be compromised for most of the dimensions of anxiety.

## Developing More Refined Instruments for Measuring Clinical Variables

The majority of the contributions was related to the development and refinement of psychological tests using a transcultural approach. Some Authors presented national adaptation of questionnaires assessing emotional regulation in clinical and non-clinical populations (“Assessment of Affect Lability: Psychometric Properties of the ALS-18,” “Psychometric properties of the Cognitive Emotion Regulation Questionnaire (CERQ) in patients with fibromyalgia syndrome”; “Confirmatory Factor Analysis of the French Version of the Savoring Beliefs Inventory”).

Other papers were illustrative of the psychometric functioning of questionnaires assessing dispositional traits, such as the capacity to love (“Measuring the Capacity to Love: development of the CTL-Inventory,” “Italian validation of the Capacity to Love Inventory: preliminary results”), that is an important diagnostic marker in clinical contexts (e.g., in pathological narcissism), was and a significant outcome parameter of psychotherapeutic treatment; the intolerance of uncertainty (“The Intolerance of Uncertainty Inventory: validity and comparison of scoring methods to assess individuals screening positive for anxiety and depression”), which was found to be associated with a difficulty to tolerate absence of sufficient information and sustain the perception of uncertainty (Carleton, 2016a,b); the expectations correlated with selfies-taking and posting in adolescents (“Selfie expectancies among adolescents: construction and validation of an instrument to assess expectancies toward selfies among boys and girls”); or assessing the cognitive self-defeating schemas (“Early Maladaptive Schemas” conceptualized by Young et al., 2003), associated with the development of personality disorders and many axis-I disorders (“Psychometric properties of the Italian version of the Young Schema Questionnaire L-3: preliminary results”).

Particularly, instruments such as the CTL-Inventory (Kapusta et al.), composed of six dimensions of the human disposition to establish relationships strictly connected to a person's psychic development, yields good internal consistency with stable and consistent results in three culturally different (Austrian, Poland, and Italian) samples, and very good test–retest reliability, as well as negative associations with depression, narcissism and promiscuity, and positive associations with relationship qualities such as conflicts, support and depth. Correlated with this disposition, in the paper “Measuring intimate partner violence and traumatic affect: development of VITA, an Italian scale” the author proposed an interesting self-report questionnaire (VITA Scale: Intimate Violence and Traumatic Affects Scale) for measuring intensity of post-traumatic affect, derived from intimate partner violence, the most widespread form of violence against women (World Health Organization [WHO], 2013).

Finally, “The reliability of the DEM test in the clinical environment” paper represents an example of adaptation of psychological test with medical outcome using a transcultural approach. The developmental eye movement (DEM) test could represent a practical and easy method for assessing and quantifying ocular motor skills and evaluating performance over time in children in clinical settings.

One of the common issues for practitioners or those using self-report inventories of personality and psychopathology concerns the susceptibility to malingering or faking. In the “Could time detect a faking-good attitude? A study with the MMPI-2-RF” paper, the authors addressed the role of time in detecting the intentional and deliberate behaviors that helps an individual achieve personal goals (Faking-Good attitude).

## Evaluating Methodological Issues Involved into Therapeutic Outcomes and Processes

Reflecting state-of-the-art scientific literature, all the papers described above are based on the classical test theory (CTT; Spearman, 1904; Novick, 1965; Gulliksen, 2013). The CTT relies on the evaluation of the reliability, validity, and factor structure of a defined psychological measure (e.g., Innamorati et al., 2013, 2014b, 2015), but within this framework it is impossible to distinguish and compare the parameters related to the individuals (abilities or traits or clinical dimensions, such as depression, anxiety; e.g., Balsamo, 2013; Balsamo and Saggino, 2014; Balsamo et al., 2014) and those relative to the items (difficulties).

Two additional papers presented important contributions from two different methodological frameworks, the Item Response Theory (IRT; Rasch, 1960; Lord, 1980), and the Formal Psychological Assessment (FPA; Spoto, 2011; Spoto et al., 2013).

IRT has been found to offer a useful approach to address some drawbacks of the CTT-based instruments (e.g., to develop new assessment measures to use in psychiatric settings; to shorten full-length tools or refine existing instruments, to address content redundancy). In the paper “Using Item Response Theory for the Development of a New Short Form of the Eysenck Personality Questionnaire-Revised,” the IRT was used to develop a new version of a short form of the Eysenck Personality Questionnaire-Revised (EPQ-R), which includes Psychoticism, Extraversion, Neuroticism, and Lie scales. It outperformed the original instrument (EPQ-R; Eysenck et al., 1985), providing further evidence toward the usefulness of assessing personality traits in clinical settings via IRT.

One intriguing IRT feature concerns the ability to detect respondents in the faking condition from those in the sincere condition. In the study “Using overt and covert items in self-report personality tests: susceptibility to faking and identifiability of possible fakers”, a one-parameter Rasch model, Rasch, 1960; Andrich, 1988) was applied for analyzing items of the alexithymia scale categorized as overt or covert by expert psychotherapists in order to investigate the influence of faking on overt and covert items, and to identify these possible fakers.

An interesting perspective in the assessment of emotional psychopathology was provided by authors of the paper “New perspectives in the adaptive assessment of depression: the ATS-PD version of the QuEDS.” They proposed an Adaptive Testing System for Psychological Disorders (ATS-PD) version of the Qualitative-Quantitative Evaluation of Depressive Symptomatology questionnaire (QuEDS). Adaptive testing could be used to shorten questionnaires without loss of information, reducing the assessment time and focusing on the specific clinical configuration presented from the patients (Petersen et al., 2006).

## Conclusion

Scientists were invited to submit contributions that could facilitate sharing of knowledge among clinicians and researchers engaged in the metric evaluation of clinical phenomena. The ultimate goal is to disseminate a culture of the integration between “psychometric model” and “clinical model,” promoting the scientific debate about the enhancement of the existing methods and/or the proposal of new methods capable of combining clinical significance with the quantitative rigor (Balsamo, 2010; Balsamo et al., 2015c).

Much work needs to be done, but some major issues have been raised by several authors committed to this discipline and have some answers have been obtained in this Research Topic. The response to the call for papers yielded a wealth of proposals with 19 accepted papers by 92 contributing authors.

Our Research Topic included important studies which provide a state-of-the-art scientific compendium of recent and sound psychometric tools useful for improving evidence-based procedures. To the extent that we managed to counter the widespread tendency of the research in clinical psychology and psychiatry to persevere in using inadequate measurement instruments for the diagnosis of disorders and the evaluation process of treatment, we have attained the goal we set ourselves. Only in this way, results derived from clinical research will be no more purely formal and academic, but will have a significant impact on patients' well-being (Nierenberg and Sonino, 2004).

To our delight, several of the articles included have already been accessed thousands of times, indicating a genuine interest in the topics covered.

## Author Contributions

All authors listed have made a substantial, direct and intellectual contribution to the work, and approved it for publication.

### Conflict of Interest Statement

The authors declare that the research was conducted in the absence of any commercial or financial relationships that could be construed as a potential conflict of interest.
